# Comprehensive analysis reveals the potential value of inflammatory response genes in the prognosis, immunity, and drug sensitivity of lung adenocarcinoma

**DOI:** 10.1186/s12920-022-01340-7

**Published:** 2022-09-18

**Authors:** Congkuan Song, Shize Pan, Donghang Li, Bo Hao, Zilong Lu, Kai Lai, Ning Li, Qing Geng

**Affiliations:** Department of Thoracic Surgery, Renmin Hospital of Wuhan University, No.238 Jiefang Road, Wuchang District, Wuhan, 430060 China

**Keywords:** Lung adenocarcinoma, Inflammation, Tumor microenvironment, Prognosis, Therapeutic responses

## Abstract

**Background:**

Although the relationship between inflammatory response and tumor has been gradually recognized, the potential implications of of inflammatory response genes in lung adenocarcinoma (LUAD) remains poorly investigated.

**Methods:**

RNA sequencing and clinical data were obtained from multiple independent datasets (GSE29013, GSE30219, GSE31210, GSE37745, GSE42127, GSE50081, GSE68465, GSE72094, TCGA and GTEx). Unsupervised clustering analysis was used to identify different tumor subtypes, and LASSO and Cox regression analysis were applied to construct a novel scoring tool. We employed multiple algorithms (ssGSEA, CIBERSORT, MCP counter, and ESTIMATE) to better characterize the LUAD tumor microenvironment (TME) and immune landscapes. GSVA and Metascape analysis were performed to investigate the biological processes and pathway activity. Furthermore, ‘pRRophetic’ R package was used to evaluate the half inhibitory concentration (IC50) of each sample to infer drug sensitivity.

**Results:**

We identified three distinct tumor subtypes, which were related to different clinical outcomes, biological pathways, and immune characteristics. A scoring tool called inflammatory response gene score (IRGS) was established and well validated in multiple independent cohorts, which could well divide patients into two subgroups with significantly different prognosis. High IRGS patients, characterized by increased genomic variants and mutation burden, presented a worse prognosis, and might show a more favorable response to immunotherapy and chemotherapy. Additionally, based on the cross-talk between TNM stage, IRGS and patients clinical outcomes, we redefined the LUAD stage, which was called ‘IRGS-Stage’. The novel staging system could distinguish patients with different prognosis, with better predictive ability than the conventional TNM staging.

**Conclusions:**

Inflammatory response genes present important potential value in the prognosis, immunity and drug sensitivity of LUAD. The proposed IRGS and IRGS-Stage may be promising biomarkers for estimating clinical outcomes in LUAD patients.

**Supplementary Information:**

The online version contains supplementary material available at 10.1186/s12920-022-01340-7.

## Background

As the most common subtype of non-small cell lung cancer (NSCLC), lung adenocarcinoma (LUAD) has its complex oncogenic mechanisms and heterogeneity [1–5]. This is an important reason why cancer patients at the same stage have different clinical outcomes and show different responses to the same drug treatment. Even with such medical progress, the prognosis judgment and treatment of LUAD are still challenging. In recent years, a large number of studies [4–9] have attempted to construct classifiers for prognostic risk stratification and drug response prediction in NSCLC patients. Unfortunately, most of the prognostic signatures proposed in these studies still face some limitations of routine clinical practice. In the era of precision medicine, a reliable prognostic stratification system is urgently needed to optimize patients’ prognosis prediction and treatment decision-making.

Inflammatory microenvironment is considered as a hallmark of cancer, and an increasing number of studies have gradually confirmed the effect of the inflammatory response on tumorigenesis and progression [10–16]. TNF-a has been reported to alter the tumor microenvironment (TME), enhance tumor aggressiveness, and promote metastasis [14]. Macrophages represented a significant portion of immune infiltrate in most cancers and M1 pro-inflammatory macrophages was also considered as anti-tumor cells [15]. Meanwhile, the CXCL1/CXCR2 signaling pathway was also thought to play important roles in regulating tumor growth and promoting tumor metastasis [16]. However, although the relationship between inflammatory response and tumor has been gradually recognized, there is still a lack of comprehensive analysis of inflammatory response genes (IRGs) in prognosis, immunity and drug therapy of LUAD.

In this study, we comprehensively analyzed the IRGs and identified three distinct tumor subtypes with obviously different clinical outcomes and immune characteristics in LUAD. Besides, based on IRGs, we set up a scoring tool called inflammatory response gene score (IRGS), which was also strongly correlated with immune infiltration and genomic landscape in LUAD and displayed the potential in predicting drug therapeutic responses. These findings suggested that the IRGs played non-negligible roles in shaping individual tumor immune microenvironment. Deep understanding of the multifaceted significance of IRGs in LUAD facilitates more rational intervention strategies for LUAD patients.

## Materials and methods

### Data download and pre-processing

First, we obtained 200 IRGs from the GSEA website (http://www.gsea-msigdb.org/gsea/index.jsp), and LUAD transcription profile data and clinical information from TCGA (https://portal.gdc.cancer.gov/), GEO (https://www.ncbi.nlm.nih.gov/geo/). We also obtained 288 lung normal tissues from the Genomic Tissue Expression (GTEx) database (https://commonfund.nih.gov/GTEx/) to obtain adequate lung normal tissue matching the tumor tissue. In total, eight LUAD-independent datasets from GEO were included in this study, all containing patient survival data. These datasets were respectively, GSE29013 [17], GSE30219 [18], GSE31210 [19], GSE37745 [20], GSE42127 [9, 21], GSE50081 [7], GSE68465 [22] and GSE72094 [23]. For these datasets (GSE29013, GSE30219, GSE31210, GSE37745, and GSE50081) from the same chip platform, we used the "combat" algorithm of "SVA" R package to integrate them into a new cohort (meta-cohort) to reduce the batch effect caused by non biotechnology bias [24, 25]. In addition, we also obtained the somatic mutation data in LUAD from the TCGA and the copy number variation (CNV) data from the UCSC Xena (https://xenabrowser.net).

### Identification of the candidate genes and unsupervised clustering analysis

The ‘limma’ R package was used to explore the significantly differentially expressed genes (DEGs) between lung tumor and normal tissues based on TCGA and GTEx databases. The cut-off value was |log2FC|> 1 and FDR < 0.05 (FC, fold change; FDR, false discovery rate). After integrating the transcriptional profiling data and survival data, we performed univariate Cox analysis on IRGs. Subsequently, unsupervised clustering analysis was applied to identify distinct tumor molecular subtypes based on the expression of these candidate genes (*p* < 0.05 in univariate Cox analysis) and classify patients for further analysis. We used the ‘ConsensuClusterPlus’ package [26] to perform the above steps and 1000 times repetitions were conducted for guaranteeing the stability of classification.

### Establishment and validation of an inflammatory response gene score system

We performed the LASSO analysis and multivariate Cox analysis for the selected genes acquired above, and calculated the coefficients of the genes. Based on gene coefficients and expression values, we calculated the inflammatory response gene score (IRGS) for each sample using the following formula:

IRGS = β1 × expressionG1 + β2 × expressionG2 + …β***n*** × expressionG***n***. To validate the predictive performance of the scoring system constructed in this study, we assessed survival differences between subgroups using the Kaplan–Meier survival analysis (log-rank test), and plotted the receiver operating characteristic (ROC) curves compared with previously developed signatures [27–30]. Furthermore, to validate the predictive performance of the IRGS in the different LUAD subgroups, we also performed a subgroup analysis.

### Estimation of the immune characteristics

To comprehensively characterize the TME and immune landscapes of LUAD, we used multiple algorithms. Single sample GSEA (ssGSEA) [31], CIBERSORT [32], and MCP counter [33] were applied to quantify the infiltration abundance of various immune cells. The Estimation of Stromal and Immune Cells in Malignant Tumors using Expression Data (ESTIMATE) algorithm [33], was employed to infer tumor purity and calculate the immune and stromal scores.

### Gene set variation analysis (GSVA) and functional annotation

We downloaded the ‘h.all.v7.4.symbols’ (GSEA hallmark sets) from MSigDB (http://www.gsea-msigdb.org/gsea/msigdb). Subsequently, we used GSVA method [34] to estimate the difference on pathway activity between different subgroups. The cut-off value was set as |log2FC|> 0.1 and adj. *p* value < 0.05. We also performed a pathway enrichment analysis of the related genes using KEGG [35]

### Estimation of drug sensitivity

Half inhibitory concentration (IC50) are widely used to assess drug efficacy. In this study, we used the ‘pRRophetic’ R package [36] to evaluate the IC50 of each sample to infer drug sensitivity. Additionally, these files named “RNA: RNA-seq” and “Compound activity: DTP NCI-60”were also obtained from the CellMiner databases (https://discover.nci.nih.gov/cellminer/home.do). We further investigated the pearson correlation of the drug IC50 with the expression values of IRGs. The selected IRGs were used as the receptor and the corresponding drugs as the ligand to docking the active components (compounds) and the corresponding targets through Vina software. The compounds were then dehydrated and removed from the original ligand by the PyMOL 2.4.0 software, and the results were visually analyzed using Discovery Studio.

### Additional bioinformatic and statistical analyses

All scores including checkpoint genes, immune inhibitors, immune stimulators, tumor infiltrating lymphocytes (TILs), IFN response, cytolytic activity (CYT), and HLA were calculated based on the ssGSEA algorithm using the corresponding gene sets (Additional file 2: Table S1). We calculated the tumour immune dysfunction and exclusion (TIDE) score [37] for each sample of the LUAD by using an online tool (http://tide.dfci.harvard.edu). Based on it, we can predict the potential response to immunotherapy in different subgroups of patients. All statistical analyses were done in R 3.6.2 software, and *p* < 0.05 was considered statistically significant.

## Results

### Differentially expressed IRGs and functional annotation

**Fig. 1:**
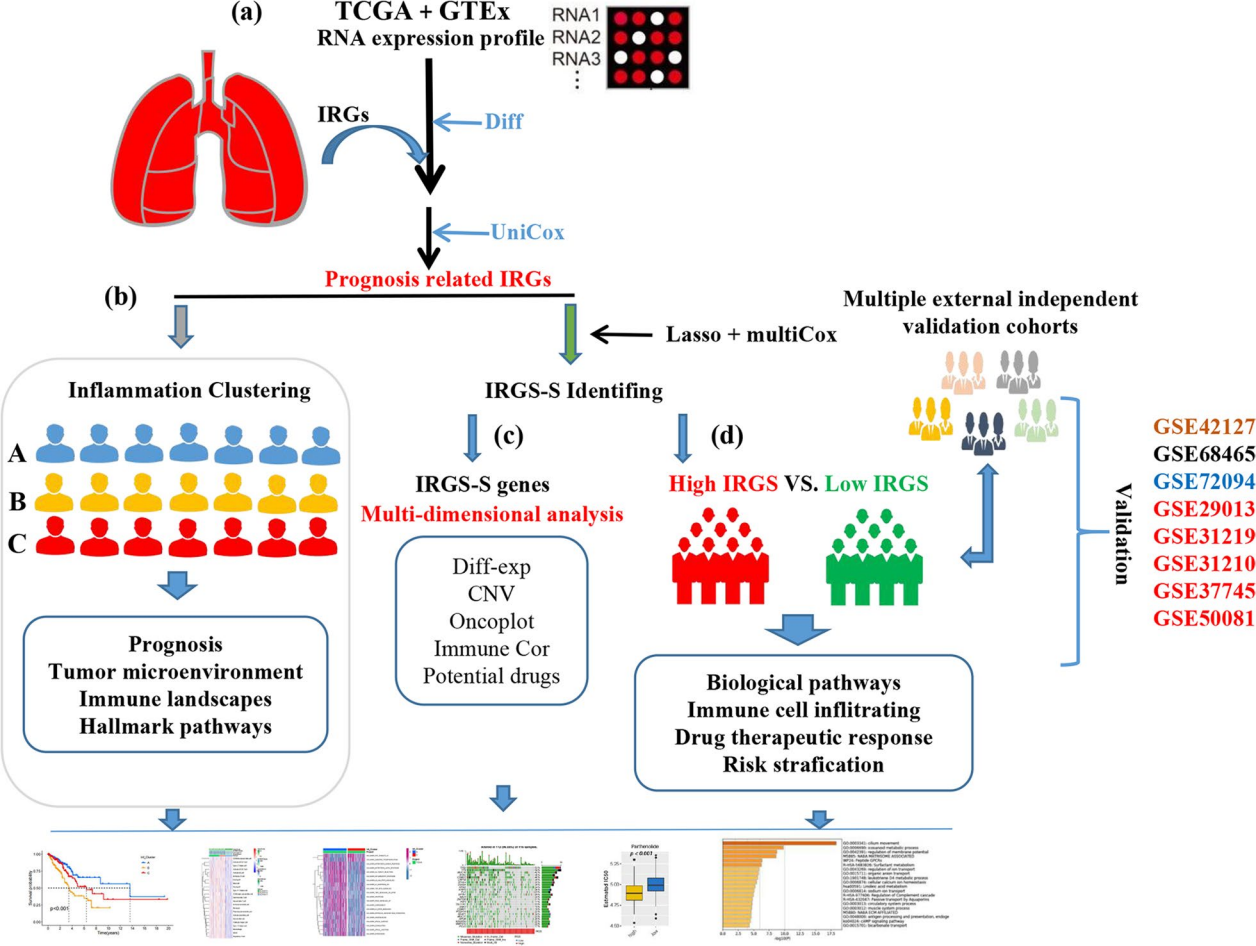
The workflow and content of this study. This flow chart consists of four parts: **a**, **b**, **c**, **d**, described as follows: **a** Identification of prognostic related IRGs; **b** Unsupervised clustering to identify different LUAD subtypes; **c** Multi-dimensional characterization of IRGS-S genes **d** Multifaceted differences between high and low IRGS subgroups

The workflow and content of this study are shown in Fig. 1. This flow chart consists of four parts: a, b, c, d, described as follows: (a) Identification of prognostic related IRGs; (b) Unsupervised clustering to identify different LUAD subtypes; (c) Multi-dimensional characterization of IRGS-S genes (d) Multifaceted differences between high and low IRGS subgroups. Of the 200 IRGs obtained from the GSEA website, 139 genes differed in expression between tumor and normal tissues. Among them, 67 genes were up-regulated in tumor tissues, and 72 genes were down-regulated. Additional file 1: Fig. S1a, b visualized the top 5 pathways significantly enriched for up- and down-regulated genes in LUAD, respectively. Among them, the three pathways that up-regulated genes are mainly involved in are cytokine-cytokine receptor interaction, viral protein interaction with cytokines and cytokine receptors, and chemokine signaling pathway, while the three pathways that down-regulated genes are mainly involved in are TNF signaling pathway, Yersinia infection, and JAK-STAT signaling pathway. All of these pathways are significantly linked to the host inflammatory response.

### Three distinct tumor subtypes revealed different prognosis, biological processes, and immune characteristics in LUAD

**Fig. 2:**
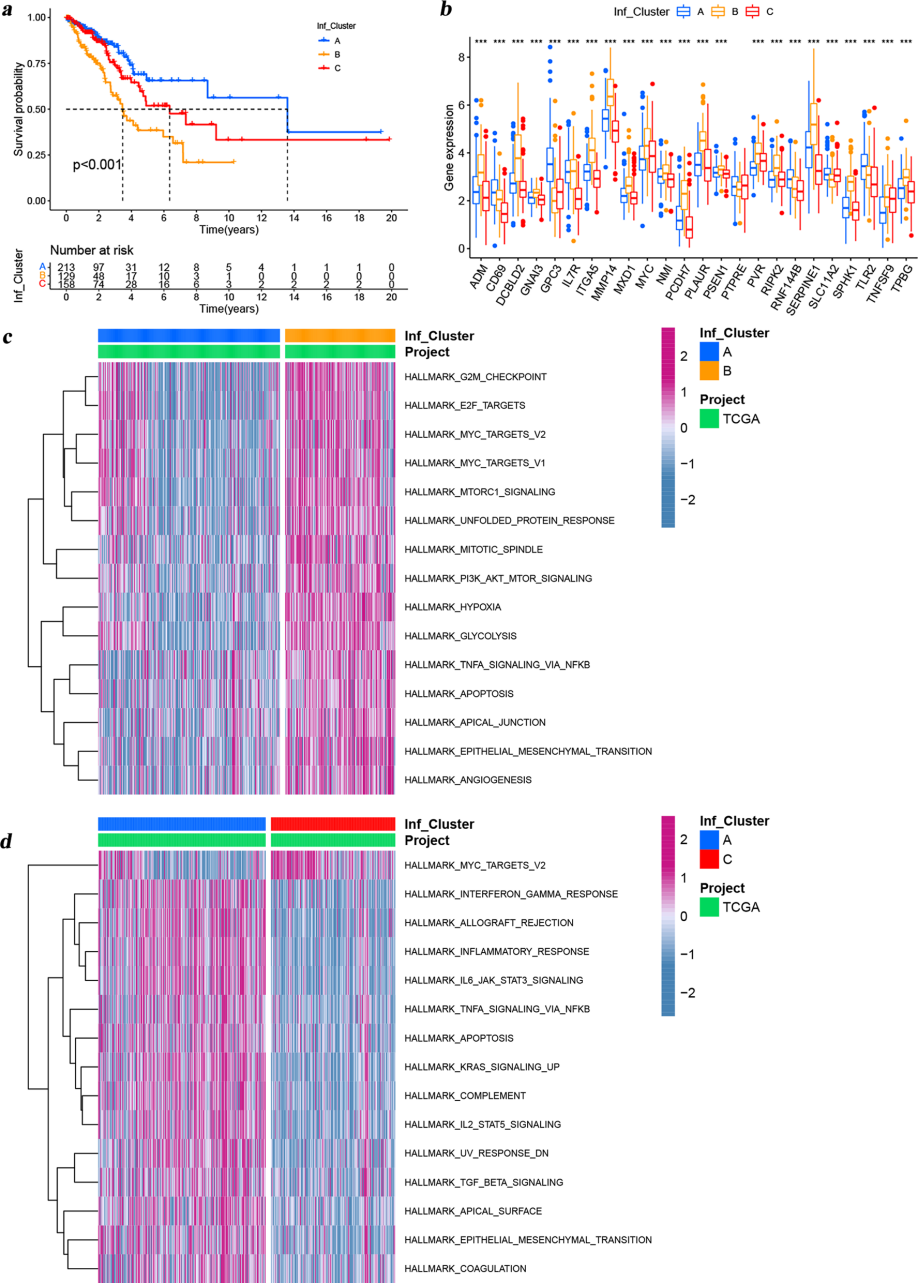
Differences in prognosis, IRG RNA expression and enriched pathways among the three distinct tumor molecular subtypes. **A** Kaplan–Meier **s**urvival analyses for the three tumor molecular subtypes based on 500 patients from TCGA-LUAD cohort including 213 cases in Inf-Cluster A, 129 cases in Inf-Cluster B and 158 cases in Inf-Cluster C. **B** Expression patterns of 24 prognosis-related IRGs among distinct tumor molecular subtypes. **C**, **D** GSVA enrichment analysis showing the activation states of biological pathways in distinct tumor molecular subtypes. The heatmap was used to visualize these biological processes, and MediumVioletRed represented activated pathways and SteelBlue represented inhibited pathways. The TCGA-LUAD cohort was used as a sample annotation. **C**: Inf-Cluster A vs B; **D**: Inf-Cluster C vs A

Univariate Cox analysis revealed a close association with prognosis in 24 of the 139 differentially expressed IRGs (Additional file 1: Fig. S1c). The 24 prognosis-related IRGs probably played critical roles in the formation of different tumor subtypes, and were implicated in cancer pathogenesis and progression. To testify these hypotheses, we applied the “ConsensusClusterPlus” R package to perform unsupervised clustering of LUAD patients based on the transcriptional profiling data of these 24 prognosis-related IRGs for classifing patients with qualitatively different subtypes, and three distinct tumor molecular subtypes were eventually identified (Additional file 1: Fig. S2a, b), including 213 cases in subtype A, 129 cases in subtype B and 158 cases in subtype C. We termed these three subtypes as Inf-Cluster A, Inf-Cluster B, Inf-Cluster C, respectively, among which Inf-Cluster A presented a prominent survival advantage, whereas Inf-Cluster B exhibited the worst prognosis (Fig. 2a). Additionally, we also observed obvious difference in the expression of 24 prognosis-related IRGs among distinct tumor molecular subtypes. CD69, GPC3 and TLR2 were significantly elevated in the Inf-Cluster A subtype. ADM, NMI, GNAI3, PSEN1, MMP14, MXD1, DCBLD2, MYC, PCDH7, ITGA5, PLAUR, SERPINE1, RIPK2, PVR, SPHK1 and TPBG were markedly increased in the Inf-Cluster B subtype (Additional file 1: Figs. S1d and S2b). Surprisingly, the genes highly expressed in the Inf-Cluster B subtype were all prognostic risk genes (HR > 1), which could serve as an important aspect in explaining that the Inf-Cluster B subtype presented the worst prognosis.

**Fig. 3:**
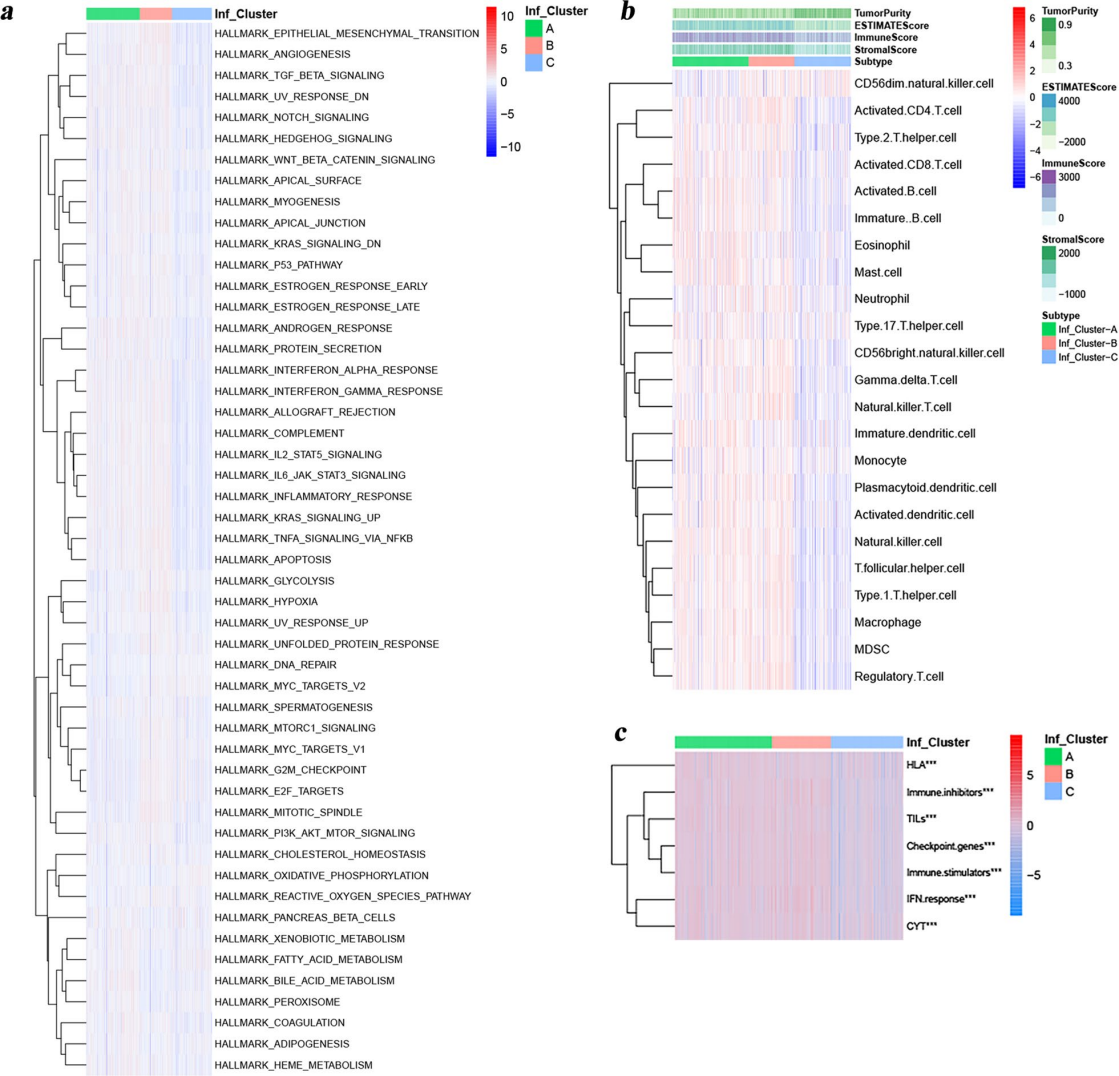
TME characteristics and immune landscapes under the three distinct tumor molecular subtypes. **A** Heatmap shows the ssGSEA score of representative Hallmark pathways curated from MSigDB in the three distinct tumor molecular subtypes. **B** Immune infiltration characteristics of the three tumor molecular subtypes based on ssGSEA algorithm. Tumor purity, ESTIMATE, immune and stromal scores from ESTIMATE algorithm are shown in annotations above. **C** Heatmap shows the ssGSEA score of representative immune signatures curated from other publications in the three distinct tumor molecular subtypes. The asterisks represented the statistical *p* value (*p < 0.05; **p < 0.01; ***p < 0.001). For comparisons of the three groups, the Kruskal–Wallis test was used

To explore the biological behaviors among these distinct subtypes, we performed GSVA enrichment analysis and ssGSEA analysis. As shown in Fig. 2c, d, Additional file 1: Fig. S2c and Additional file 3: Table S2, there were 25 hallmark pathways significantly enriched in the Inf-Cluster B subtype, which were associated with inflammatory responses as well as the important cell vital activity and metabolism, such as IL2-STAT5 signaling, epithelial mesenchymal transformation, IL6-JAK-STAT3 signaling, glycolysis, inflammatory response, interferon gamma response, PI3K-Akt-mTOR signaling, TGF-Beta signaling, apoptosis, hypoxia and so on. This result was consistent with that from ssGSEA (Fig. 3a). These pathways were markedly enriched in the Inf-Cluster B subtype and might also be important reasons for their poor prognosis. The TME affects the immune status of tumor patients and in turn affects its response to immunotherapy. Thus, ssGSEA analysis was further performed to explore the relative abundance of 23 immune-infiltrating cells in the 3 subtypes (Additional file 1: Fig. S2d and Fig. 3b). This result indicated that both some anti-tumor cells and immunosuppressive cells had significant infiltration in the Inf-Cluster B subtype, including activated CD4 + T cells, gamma delta T cells, MDSC, macrophage, NK T cells, neutrophilna, regulatory T cell, T follicular helper cell, Type 1 T helper cells and Type 2 T helper cells. Of these 23 immune cells, the vast majority presented a lower infiltration in the Inf-Cluster C subtype compared with the Inf-Cluster A and B subtypes. Moreover, further analyses by the ESTIMATE algorithm (Figs. 3b) revealed that Inf-Cluster C exhibited the lowest immune scores and highest tumor purity, while no significant differences were observed in tumor purity and immune scores between the Inf-Cluster A and B subtypes. This suggested that Inf-Cluster C subtype tumors might be surrounded by more non-tumor components. Subsequent immune signature analysis indicated that Inf-Cluster C exhibited the lowest scores in all immune signatures, while no significant differences were observed in that between the Inf-Cluster A and B subtypes (Fig. 3c). Taken together, it's not hard to find that there were no obvious differences in immune landscapes between the Inf-Cluster A and B subtypes, but their prognosis were completely different, suggesting that these immune signatures might more greatly affect the immune status of the host, but not or slightly affect the prognosis. Overall, the three distinct molecular subtypes revealed different prognosis, biological processes, and immune characteristics.

### The underlying expression perturbations and biological pathway activity across these phenotypes

Although the consensus clustering algorithm based on 24 prognosis-related IRGs classified LUAD patients into three different tumor molecular subtypes, the underlying expression perturbations and biological pathway activity across these phenotypes remained enigmatic. Thus, we further investigated the potential tumor molecular subtype-related transcriptional expression change in LUAD. We used the empirical Bayesian approach [38] to determine the shared DEGs across the 3 subtypes, and identified 162 phenotype-related DEGs using ‘limma’ R package (Additional file 4: Table S3 and Additional file 1: Fig. S3a). Subsequently, KEGG enrichment analysis revealed that these genes were primarily involved in these activities such as focal adhesion, phagosomes, NOD-like receptor signaling, and regulation of the actin cytoskeleton (Additional file 1: Fig. S3b). The results of the Gene Ontology analysis showed that the biological processes involved in DEGs included extracellular structure organization, extracellular matrix organization and phagocytosis (Additional file 1: Fig. S3c). From these results, it is not difficult to find that the biological pathways involved in these overlapping DEGs are associated with the inflammatory processes in the organism, indicating these genes could be regarded as the inflammation-related gene signatures.

### Reliable evaluation performance of the IRGS-S for LUAD prognosis prediction

**Fig. 4:**
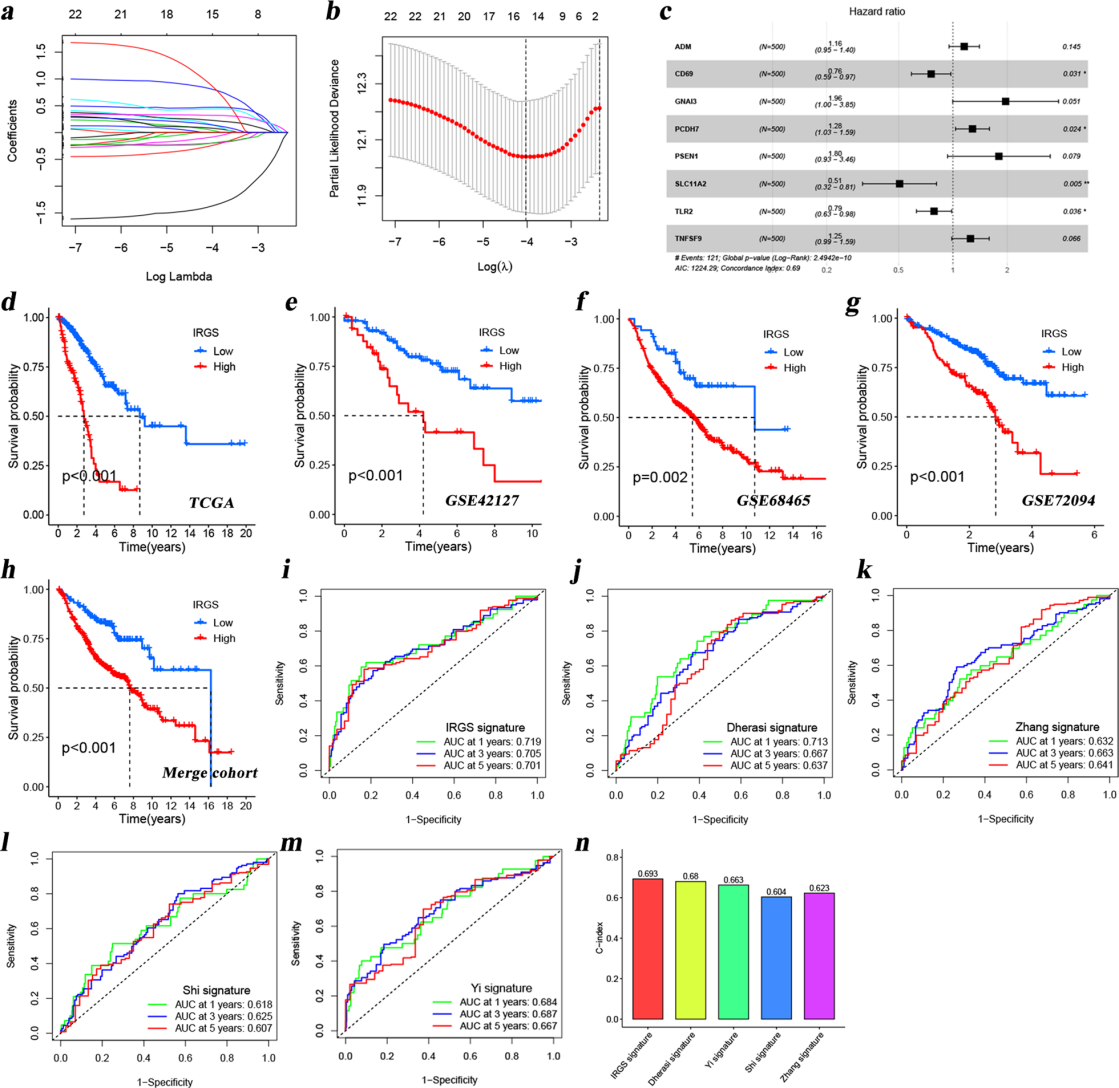
Construction and verification of an IRGS-S for LUAD patients. **A-B** These plots visualize the Lasso regression process of the 24 prognostic-correlated IRGs. **C** A forest plot reflecting a multivariate Cox regression analysis of the candidate genes in the TCGA. **D**–**H** Theses Kaplan–Meier survival curves revealed the OS differences between high- and low-IRGS groups in the TCGA **D**, GSE42127 **E**, GSE68465 **F**, GSE72094 **G** and merge-GEO **H** cohorts. GSE29013, GSE30219, GSE31210, GSE37745, and GSE50081 were from a same chip platform and thus integrated to a new cohort (meta cohort) using the ‘ComBat’ algorithm of ‘sva’ R package to reduce the batch effects from non-biological technical biases. **I**–**M** The ROC curves of the IRGS-S **I**, the sigantures from Al-Dherasi et al. **J**, Zhang et al. **K**, Shi et al. **L**, Yi et al. **M**. **N** Comparison of the C-index for each model

Based on the above findings, we believed that the three tumor subtypes based on these 24 prognostic-related IRGs could better identify patients with different prognosis as well as the immune infiltration landscapes. Thus, to further investigate the potential value of these 24 prognostic-related IRGs in LUAD, we subsequently included them in the Lasso regression analysis (Figs. 4a, b), 15 genes (ADM, GNAI3, CD69, IL7R, DCBLD2, ITGA5, RIPK2, NMI, SLC11A2, PVR, RNF144B, PCDH7, TLR2, PSEN1 and TNFSF9) were further included in the multivariate Cox analysis (stepwise regression) after filtering part of the genes. Finally, eight genes (ADM, GNAI3, PCDH7, CD69, PSEN1, SLC11A2, TLR2, TNFSF9) were included in a predictive signature (we call this the IRGS-S) (Fig. 4c and Additional file 5: Table S4). The IRGS for each patient was obtained by the following formula: IRGS = (0.144 × ExpressionAMD) + (-0.277 × ExpressionCD69) + (0.673 × ExpressionGNAI3) + (0.250 × Expression PCDH7) + (0.587 × ExpressionPSEN1) + (-0.680 × ExpressionSLC11A2) + (-0.240 × ExpressionTLR2) + (0.224 × ExpressionTNFSF9). Next, according to the optimal cut-off point of IRGS, patients were divided into high- and low-IRGS subgroups. As shown in Fig. 4d, patients with high IRGS have a worse prognosis than patients with low IRGS. This finding was also observed in four additional independent cohorts (Fig. 4e–h). In addition, the AUC values of the IRGS in our study (Fig. 4i) were also remarkably higher than that of signatures in other studies [27–30] (Fig. 4j–m). The C-index comparison of these models also reflected the same conclusions (Fig. 4n). The results from the subgroup analysis also demonstrated the good predictive performance of the IRGS-S (Additional file 1: Fig. S4), where patients with high IRGS have a worse prognosis than patients with low IRGS in many subgroups (< = 65y, > 65y, female, male, stage I/II, and stage III/IV).

### Multidimensional features underlying the IRGS-S in LUAD

**Fig. 5:**
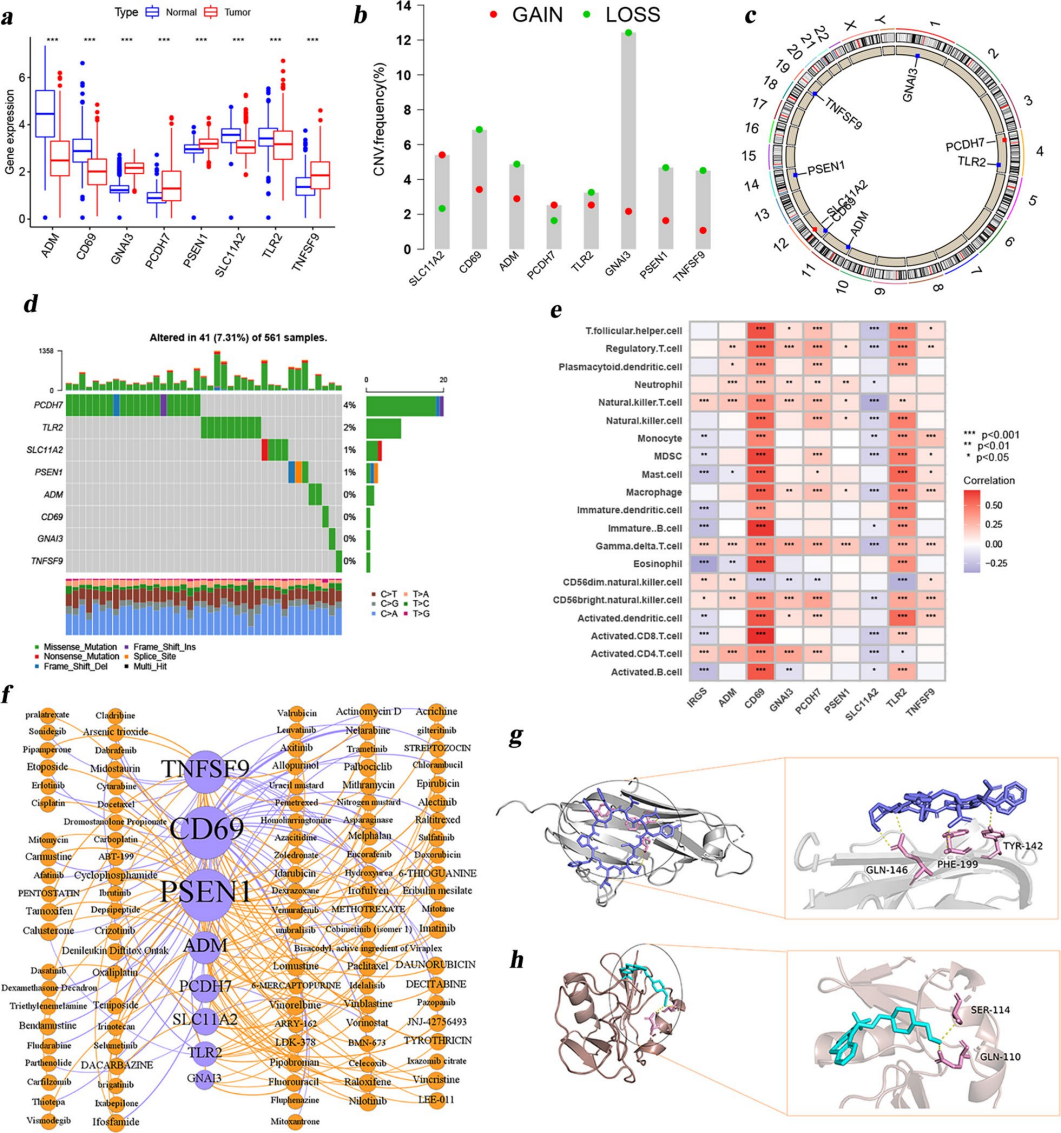
Multidimensional features of IRGS-S genes in LUAD. **A** The difference of mRNA expression levels of 8 IRGS-S genes between LUAD normal and tumor samples. **B** The bar graph showing the CNV gain/loss frequency of 8 IRGS-S genes. **C** The CNV alteration positions of these 8 IRGS-S genes on the human chromosome. **D** The waterfall plot reflecting the frequency of genetic alteration in these 8 IRGS-S genes in LUAD. **E** The plot reflecting the interrelationship between immune cells, IRGS-S genes, and IRGS. **F** Network map intuitively shows the correlation between FDA approved drugs and 8 IRGS-S genes. PSEN1 and CD69 associated with 42 and 41 drugs, respectively, with the highest proportion. The size of nodes indicates the correlation degree (number); purple lines represent positive correlation and orange represents negative correlation. The thickness of the lines represents the correlation degree (cor). **G** Docking pose and interaction of Tyrothricin with TNFSF9. **H** Docking pose and interaction of Fluphenazine with CD69

Given the significance of the IRGS-S constructed above in patient outcome prediction, we speculated that these genes included in this model had great impacts on LUAD, therefore we further revealed the multi-dimensional characteristics of these eight IRGS-S genes (ADM, CD69, GNAI3, PCDH7, PSEN1, SLC11A2, TLR2, TNFSF9) in LUAD. Differential analysis showed that GNAI3, PCDH7, PSEN1 and TNFSF9 were highly expressed in tumor tissues, while ADM, CD69, SLC11A2 and TLR2 were opposite (Fig. 5a). We also compared the expression of these genes in tumor versus normal tissues from the protein level (Additional file 1: Fig. S5). Not surprisingly, we found that the RNA expression trend of these genes was significantly different from the protein expression trend in the tumor and normal tissues. This suggests that these genes are epigenetically modified at the post-transcriptional level. CNV plays an important role in biological processes [39], intensive investigation of CNV can provide us with a new understanding of the composition of and human genome and genetic pathogenic factors. We subsequently investigated the CNV in these eight IRGS-S genes. This results revealed that CNV mutations were prevalent. ADM, CD69, GNAI3, PSEN1, TLR2, TNFSF9 showed extensive CNV deletions. In contrast, PCDH7 and SLC11A2 had prevalent CNV amplification (Fig. 5b). And the CNV alteration positions of these 8 IRGS-S genes on the chromosome were also shown in Fig. 5c. To answer the effect of CNV on gene transcription, we further investigated the correlation of CNV with gene expression. We observed that the CNV single amplification of GNAI3, PSEN1, and SLC11A2 could up-regulated the expression of the corresponding genes, while CNV single deletion of them could play the opposite effect (Additional file 1: Fig. S6c, e, f). The above results initially confirmed the tight association between CNV variants and gene expression. Further describing the somatic mutation frequency of the eight IRGS-S genes in LUAD, we found that only 41 out of the 561 samples (7.31%) experienced genetic alterations, mainly including missense mutations, nonsense mutations, and multi hit. Of the 8 IRGS-S genes, PCDH7 presented the highest mutation frequency, followed by TLR2, while ADM, CD69, GNAI3, and and TNFSF9 did not experience any mutations in LUAD (Fig. 5d). Subsequently, we also investigated the correlation between immune infiltrating cells and IRGS as well as IRGS-S genes. Surprisingly, we found that these genes were associated with at least six immune cells. Among these, CD69 was significantly associated with all 23 cells, the vast majority positively, including anti-tumor cells and immunosuppressive cells (Fig. 5e). This seemed to indicate crucial roles for IRGS genes, particularly CD69, in the TME. Additionally, as one of the key avenues of anti-tumor treatment, drugs to treat tumors have always been a hot topic of research. More and more drugs are being developed and used in clinical practice. Given the importance of these 8 IRGS-S genes in LUAD, we explored the relevance of these genes with FDA approved drugs. Figure 5f and Additional file 6: Table S5 intuitively showed the correlation between these drugs and IRGS-S genes. PSEN1 and CD69 associated with 42 and 41 drugs, respectively, with the highest proportion. The combinations with the strongest correlation were randomly selected for molecular docking, as shown in Fig. 5g, h, visualizing the Fluphenazine with CD69 and Tyrothricin with TNFSF9 docking sites. This suggested that these genes might be potential targets for drug therapy in LUAD. This provides valuable clues to the individualized treatment of LUAD patients. Globally, the above analysis illustrates the multiple traits of the eight IRGS-S genes in LUAD, including differential expression at transcription levels, genomic alterations, correlation with immune-infiltrating cells, and the effects of FDA-approved drugs. These IRGS-S genes have important implications in LUAD.

### Comprehensive analyses of enriched biological processes and immune infiltration between different IRGS subgroups

**Fig. 6:**
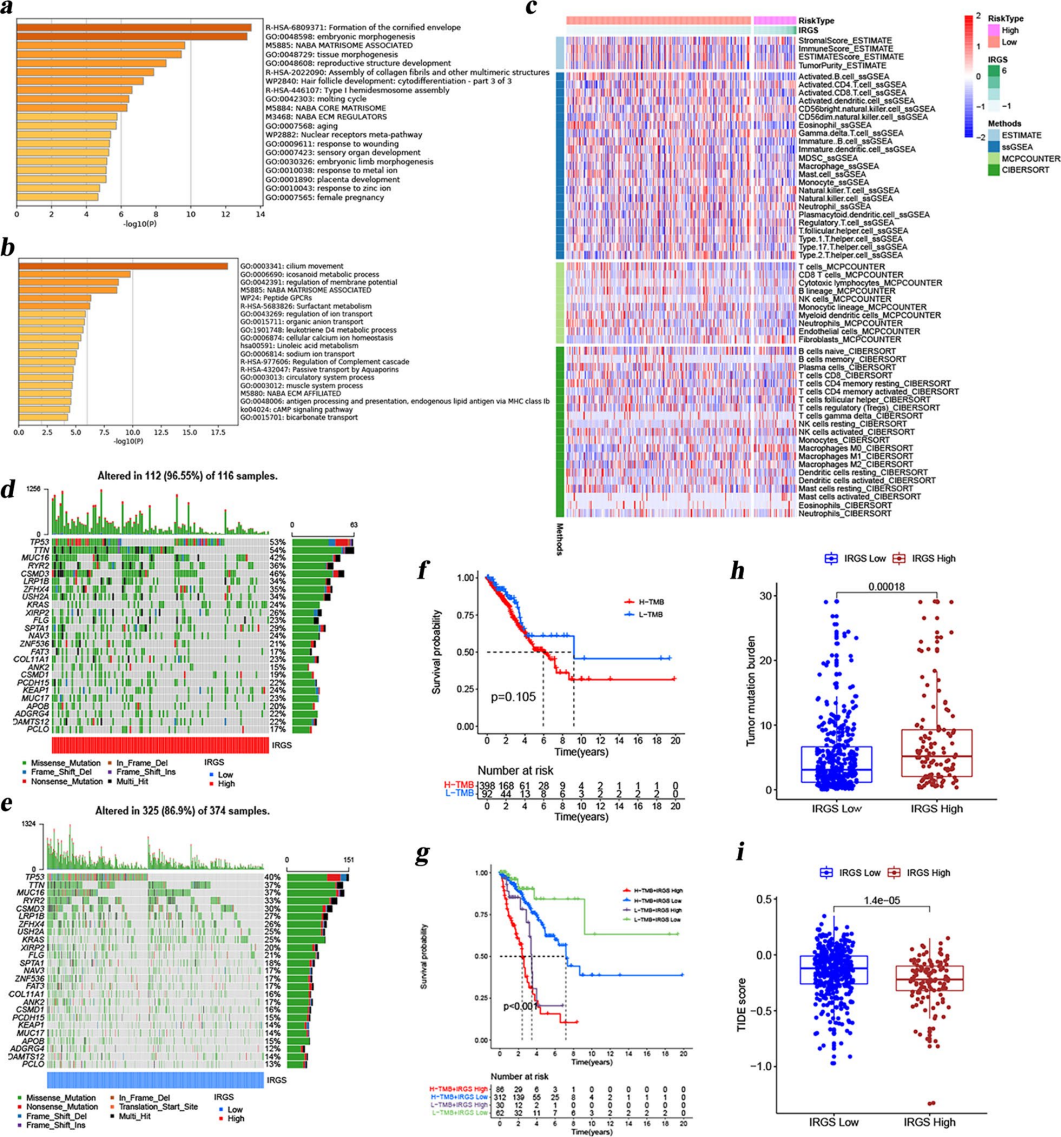
Enriched biological pathways, immune infiltration and genetic characteristics in different IRGS subgroups. **A**, **B** Metascape analysis was performed to reveal the enriched pathways that over-expressed genes **A** and low-expressed genes **B** in the high-IRGS group were mainly involved in. **C** Comparison of immune cell infiltration abundance in the high- and low-IRGS groups based on ssGSEA, CIBERSORT, and MCP counter algorithms. **D**, **E** Genetic alterations in the top 25 common tumor-mutated genes in the high **D** and low **E** IRGS groups. **F** Comparison of overall survival  in the high- and low-IRGS groups. **G** The Kaplan-Meier survival curve reflecting the interrelationship among TMB, IRGS and patient survival. **H** Comparison of TMB in the high- and low-IRGS groups. **I** Comparison of TIDE scores in the high- and low-IRGS groups

We acquired 3768 DEGs between the high and low IRGS groups (Additional file 7: Table S6). Of these, 520 genes were highly expressed in the high-IRGS group and 3248 were poorly expressed. The results from Metascape analysis revealed that these genes over-expressed in the high-IRGS group were mainly involved in formation of the cornified envelope, embryonic morphogenesis, NABA matrisome associated and so on (Fig. 6a), while these genes over-expressed in the low-IRGS group were mainly enriched in cilium movement, icosanoid metabolic process, regulation of membrane potential and so on (Fig. 6b). In view of the important role of immune cells in TME, we used multiple algorithms to quantify the abundance of immune cell infiltration, and estimated the tumor purity and immune score for each sample. Our results indicated that patients in high and low IRGS subgroups were significantly different in terms of tumor purity and immune scores, that is, high IRGS patients had higher tumor purity, whereas the immune scores were the opposite. Moreover, they also showed clear differences in immune cell infiltration, as shown in Fig. 6c. The revelation of these findings allows us to more clearly recognize the complexity of the TME. The high and low IRGS groups had significantly different enrichment pathways and TME landscapes, which might be an intrinsic mechanism leading to their significant differences in prognosis.

### IRGS as a reliable marker of drug therapeutic response in LUAD

**Fig. 7:**
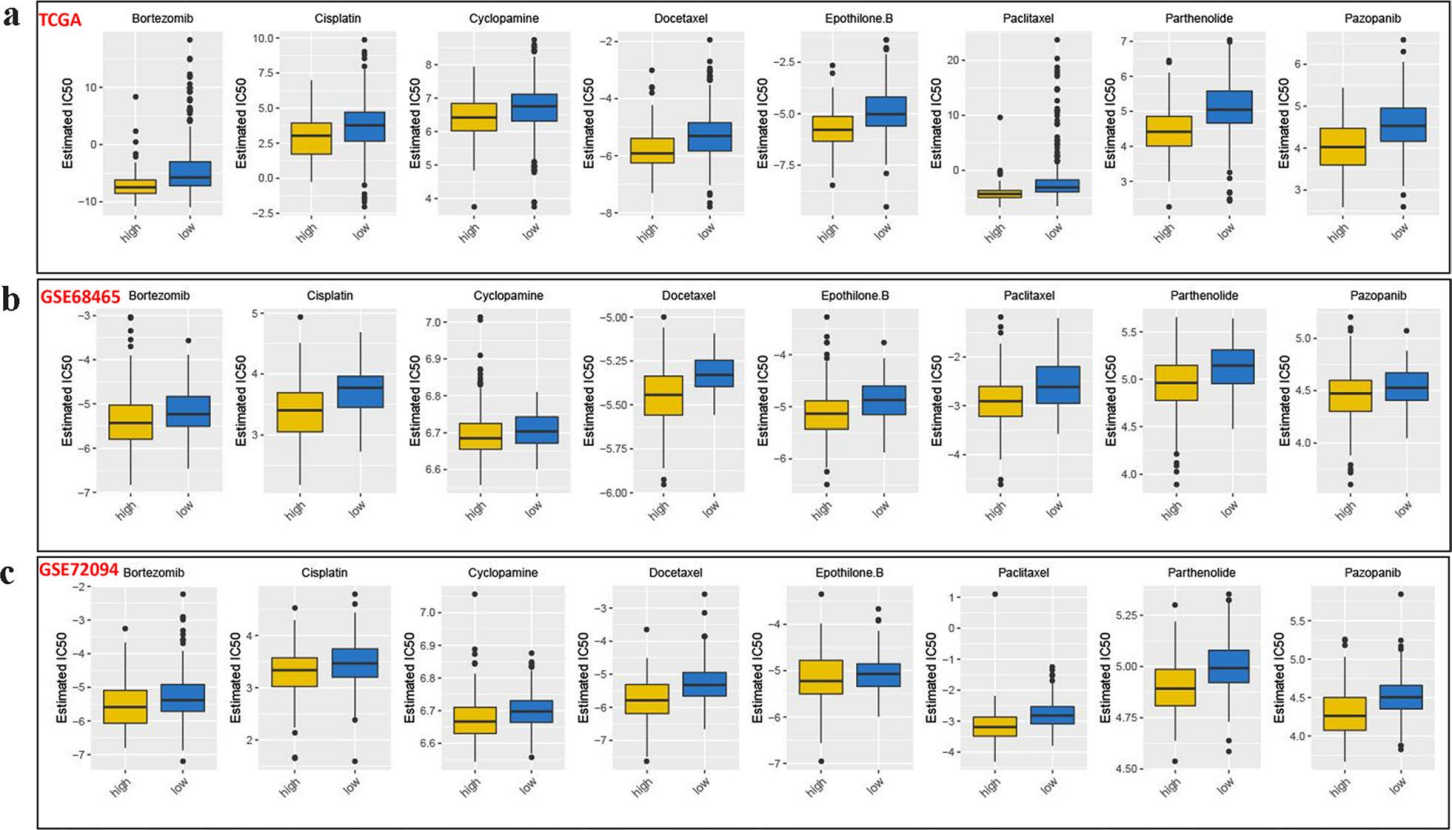
Comparison of drug sensitivities in the high- and low-IRGS groups. The estimated IC50s of Bortezomib, Cisplatin, Cyclopamine, Docetaxel, Epothilone.B, Paclitaxel, Parthenolide, Pazopanib in the high- and low-IRGS groups in the TCGA (**A**), GSE68465 (**B**), GSE72094 (**C**) cohorts

From the previous analysis, we observed clear differences in the prognosis, enriched pathways, and immune landscape between high and low IRGS patients. We were curious about differences in somatic mutations. Therefore, we characterized the somatic mutation profiles of two sets of samples based on TCGA-LUAD genomic data. From Fig. 6d, e, we found that TP53, TTN, and MUC16 were the most frequently mutated genes in LUAD. Patients with high IRGS group had a higher proportion of gene mutations. It was also not difficult to explain why patients with high IRGS presented with a higher tumor mutation burden (TMB) (Fig. 6f). Further to the survival analysis, we found that overall survival was not significantly different in patients with high and low TMB (Fig. 6g). However, patients with high TMB and high IRGS had the worst prognosis, while patients with low TMB combined with low IRGS presented the best outcomes (Fig. 6h). The correlation between TMB and response to cancer immunotherapy has been well elucidated in previous studies [40, 41]. Our study found a higher TMB in patients with high IRGS (Fig. 6f). This result seems to imply that patients with high IRGS may have a better response to immunotherapy. To consolidate this inference, we computed the TIDE score for each sample. Not surprisingly, we found that patients with high IRGS presented with lower TIDE scores (Fig. 6i). Based on these findings, we believe that patients with high IRGS may be beneficiaries of immunotherapy. Furthermore, considering that chemotherapy remains an important means of cancer therapy in clinical practice, we also evaluated the IC50 of each sample against common chemotherapeutic agents. In the analysis of the LUAD transcription profiling data from three independent datasets (TCGA, GSE68465 and GSE72094), we found that the IC50 of some common chemotherapeutic agents was lower in patients in the high IRGS group (Fig. 7a–c), indicating higher sensitivity to these drugs in patients with high IRGS. These results highlight the important value of IRGS in the prognostic stratification and drug efficacy prediction for LUAD.

### The novel LUAD staging system (IRGS-Stage) for optimizing patient outcome prediction

**Fig. 8:**
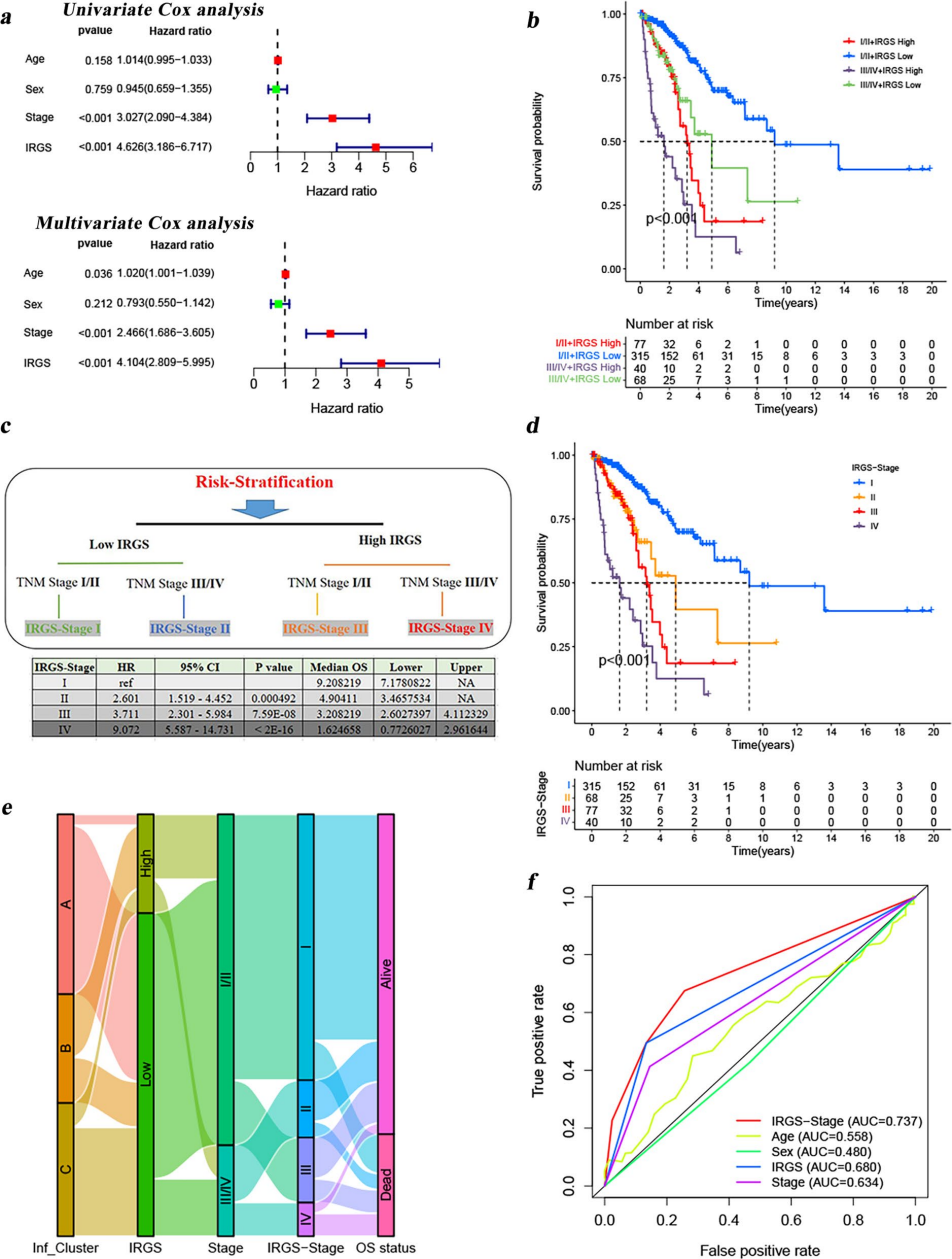
Establishment and evaluation of the novel proposed IRGS-Stage. **A** Forest map shows that IRGS is an independent prognostic predictor in LUAD by univariate and multivariate Cox analyses. **B** The cross-talk among TNM stage, IRGS and patient survival. **C** Establishment of the IRGS-Stage and risk stratification based on the cross-talk among TNM stage, IRGS and patient survival. The results of univariate Cox analysis for the IRGS-Stage are shown below the figure. **D** The Kaplan–Meier survival curves revealed remarkable differences in survival in different IRGS-Stage patients. **E** Alluvial diagram of Inf_Clusters (3 molecular subtypes) in groups with different IRGS, TNM stage, IRGS-Stage and survival status. **F** ROC curves for age, gender, stage, IRGS and IRGS-Stage

More accurate prognostic prediction was crucial for clinical treatment decisions. Therefore, we also tried to optimize the existing TNM staging system. First, we performed both a univariate and multivariate Cox analysis on 490 samples obtained from the TCGA-LUAD project. TNM stage and IRGS were identified as two independent prognostic factors, as shown in Fig. 8a. Based on the TNM stage and IRGS, we divided the patients into four categories: (1) stage I/II and low IRGS; (2) stage I/II and high IRGS; (3) stage III/IV and low IRGS; 4) stage III/IV and high IRGS. Surprisingly, we found that this classification distinguished the patient prognosis very well (Fig. 8b, c). Thus, we defined patients with low-IRGS combined with TNM-Stage I/II as IRGS-Stage I, patients with low-IRGS combined with TNM-Stage III/IV as IRGS-Stage II, patients with high-IRGS combined with TNM-Stage I/II as IRGS-Stage III, and patients with high-IRGS combined with TNM-Stage III/IV as IRGS-Stage IV (Fig. 8c). Both univariate Cox analysis (Fig. 8c) as well as K-M survival analysis (Fig. 8d) suggested significantly different prognosis in these patients with different IRGS-Stages, and the median survival times for IRGS-Stage I, II, III, and IV were 9.2, 4.9, 3.2 and 1.6 years, respectively. Another, Fig. 8e also reflected the overlap and correspondence among the tumor molecular subtypes (Inf_Cluster A-C), IRGS, TNM stage, IRGS-Stage, and patient survival status. Moreover, the ROC analyses indicted the novel IRGS-Stage proposed in this study showed a more powerful capacity for survival prediction compared to the others, with highest AUC values (Fig. 8f). We believed that the IRGS-Stage was better able to refine the patient survival prediction.

## Discussion

Due to the complex oncogenic mechanisms and heterogeneity of LUAD, it remains highly challenging to accurately predict patient prognosis and develop personalized management strategies. Inflammatory response play crucial roles in tumorigesis and progression [42, 43], however, although the relationship between inflammatory response and tumor has been widely recognized, the potential implications of of inflammatory response genes in prognosis, immunity and drug therapy of LUAD remains poorly investigated. In this study, we identified three distinct tumor molecular subtypes using unsupervised clustering, and accidentally found that the three molecular subtypes presented obviously different prognosis and immune characteristics. Besides, based on IRGs, we established a scoring tool called IRGS that could effectively identify high- and low-risk patients. Its prognostic power was also validated in four independent cohorts. The prediction performance of the model presented in this study was still not inferior to other LUAD prognostic models [27–30]. Even in some LUAD patient subgroups, the IRGS retained its predictive power to effectively distinguish between high- and low-risk patients.

The IRGS-S constructed in this study included eight genes (ADM, CD69, GNAI3, PCDH7, PSEN1, SLC11A2, TLR2, TNFSF9). Of these eight genes, four (GNAI3, PCDH7, PSEN1, TNFSF9) were highly expressed in tumor tissues and were associated with poor prognosis, while CD69, SLC11A2, and TLR2 were poorly expressed in tumor tissue and were associated with good prognosis. However, ADM expression was lower in tumor tissues but considered as a prognostic risk gene. This result was in line with the findings in ovarian cancer by Ferlini C et al.[44]. ADM (Adrenomedullin) is a gene encoding preproadm located at a single locus on human chromosome 11 [45]. Previous studies [44, 46] have found that ADM is expressed in a variety of tumor tissues and cells, and can regulate tumor cell proliferation, invasion, and metastasis, and tumor angiogenesis. In our study, ADM was also identified as a poor prognostic gene for LUAD. This finding is also similar to that of Ramachandran et al. [47, 48], where ADM was also demonstrated to promote pancreatic cancer invasion and metastasis. ADM has become an important biological target in the intervention treatment of human tumors. GNAI3 is a member of the a subunit of the G protein family. In recent years, with the deepening of cancer research, the G protein family has been confirmed to play an important role in the development of cancer. The study of Faivre et al. [49]observed that the GNAI3 protein can inhibit the migratory capacity of colon cancer cells. In contrast to this finding, Ghosh et al. [50]found that GNAI3 promotes Hela cell migration. However, in this study, we found that GNAI3 mRNA showed a high expression state in LUAD tumor tissues and was associated with poor prognosis, but it showed a completely opposite trend in protein levels. Notably, there are not many reports on the functional studies of the GNAI3-encoded protein in cancer. Combined with the existing data, the GNAI3 gene does have an impact on the migration ability of cancer cells, but the molecular mechanism is still fully unknown. How the GNAI3 gene affects the biological behavior of tumor malignancy in different cancer backgrounds remains an open question. PSEN1, localized to chromosome 14q24.3 [51], can encode Presenilin-1.It is a commonly expressed multi-transmembrane domain protein, mainly located on the endoplasmic reticulum, the Golgi apparatus, and the plasma membrane. Pathogenic mutations in the PSEN1 gene are an important cause of familial Alzheimer's disease [52], which function as the core catalytic subunit of the beta-secretase complex involved in cleavage. Previous studies [53–56] have shown that PSEN1 plays a key role in the Notch and Wnt signaling cascades and in downstream regulatory processes by processing key regulatory proteins and regulating cytosolic CTNNB1 levels. Overexpression of PSEN1 promotes peritoneal metastasis in colorectal cancer, which is thought to be associated with E-cadherin proteolysis and nuclear translocation [57]. Li et al. [58] found that Presenilin-1 enhanced the invasion and migration of gastric cancer cells without changing cell proliferation. They believed that Presenilin-1 is associated with CAJ disassembly and can drive cancer progression by triggering TCF / LEF-1 activation. PSEN1 is still poorly reported in lung tumors, and this study reveals for the first time that it is overexpressed in LUAD tumor tissues, and that its enhanced expression is correlated with a worse prognosis. From the findings of the above studies, PSEN1 appears to be used as a tumor-promoting factor. However, Presenilin-1 plays completely opposite roles in tissues such as the skin. Xia et al. [59] found that the loss of Presenilin-1 in the skin led to epidermal hyperplasia and skin tumors in adult mice. Overall, PSEN1 is still poorly studied in human tumors, and how it affects tumors in different cancers remains to be investigated. As a member of the tumor necrosis factor superfamily, TNFSF9 has received increasing attention in tumors. A previous study [60] have found that TNFSF9 is expressed on a variety of tumor cells, promoting pancreatic cancer metastasis through Wnt/Snail signaling and M2 polarization of macrophages. In colon cancer patients, TNFSF9 expression is highly upregulated in tumor tissues, and is significantly correlated with the occurrence of distant metastases in advanced disease and the shortened survival [61]. Similar findings could also be observed in a study of breast cancer [62]. Our study found that TNFSF9 showed a high expression state in LUAD and was associated with poor prognosis. Current research on TNFSF9 in the prognosis of LUAD is still inadequate and needs to be further elucidated in the future. SLC11A2 is a transmembrane iron transporter known to be involved in cellular iron uptake, and acts as a proton-dependent iron import protein of Fe 2 ^+^ [63]. It has been shown that SLC11A2 expression is upregulated in endometrial cancer, and is correlated with a better prognosis [64]. Its role in other cancers, including lung cancer, has not yet been reported. In this study, the SLC11A2 mRNA was initially poorly expressed in LUAD tumor tissues, while the protein encoded by it was highly expressed, suggesting that the SLC11A2 gene may be regulated epigenetically at the post-transcriptional level. The Toll-like receptor (TLR) is a member of the superfamily of pattern recognition receptors, which play an important role in regulating inflammatory responses, cell proliferation, and apoptosis [65]. Currently, accumulating evidence [66, 67] suggests that TLR2 is closely associated with cancer progression. Our study found low TLR2 mRNA expression in LUAD tumor tissues, while TLR2 protein was high expression. Zhang et al. [68] found that TLR2 was highly expressed in the serum of lung cancer patients and promoted the progression of lung cancer. TLR2 has also been proposed as a potential therapeutic target for LUAD in cell line studies [69]. In addition, in our study, we observed that PCDH7 exhibited the highest frequency of mutation among these eight genes. PCDH7 is one of the largest subfamily members of the cadherin family, and a previous study [70] found that PCDH7 was significantly downregulated in non-muscle-invasive bladder cancer and served as an independent predictor of non-muscle-invasive bladder cancer. PCDH7 could promote the malignant transformation of bronchial epithelial cells carrying KRAS gene mutation, while knockdown of PCDH7 inhibited the growth and metastasis of lung cancer cells with a mutated KRAS gene, suggesting that PCDH7 and KRAS had a synergistic cancer-promoting effect [71, 72]. PCDH7 could bind to the regulatory protein SET of PP2A, inhibiting PP2A activity, leading to dysregulation of negative feedback in the MAPK pathway, which in turn affected lung cancer progression [71, 73]. Also, our study found that PCDH7 was highly expressed in tumor tissues and was associated with poor prognosis, with all these findings suggesting that PCDH7 might be an important cancer-promoting factor. CD69 was a membrane surface molecule expressed after T lymphocyte activation. When activated, it could further stimulate the proliferation and activation of T cells, induce the secretion of Th1 cytokines, and indirectly kill tumors [74, 75]. Given the important roles of TME in cancer immunotherapy, we also investigated the relevance of these genes to immune-infiltrating cells. Surprisingly, we found that these genes were associated with at least six immune cells. Among these, CD69 was significantly associated with all 23 cells, the vast majority positively, including anti-tumor cells and immunosuppressive cells. This seemed to indicate crucial roles for IRGS genes, particularly CD69, in the TME. This was confirmed in the results of Mita et al. [76]. They found that CD69 plays an important role in antitumor immunity, especially in regulating the depletion of tumor-infiltrating T cells and in weakening the antitumor immune response. Our study found that CD69 presented low expression in tumor tissue and was considered a good prognostic gene. This also indirectly confirmed that CD69 might be an important tumor suppressor. From the current data, CD69 is still poorly studied in tumors. The results of this study and the findings of Mita et al. [76] all suggest that CD69 may be an important potential target for the treatment of malignant tumors. Overall, these genes all play an important role in LUAD, but their research in cancer, especially lung cancer, is still insufficient, which is the focus of future researchers.

TME is the survival environment of tumors, which plays a key role in the occurrence and development of tumors, and immune-infiltrating cells constitute an important component of the TME. The results from ssGSEA, CIBERSORT, and MCP counter algorithms uncovered clearly distinct immune infiltration was found between low- and high-IRGS groups. This is consistent with what we had expected. We also observed that significant differences on immune score and tumor purity between two subgroups. This result was consistent with our previous study [5]. Our previous study [5]also found that patients with high tumor purity tended to present shorter overall survival. In other tumors, this phenomenon is not common, or even the exact opposite, such as in colorectal cancer [77] and gliomas [78]. But it seems certain that there is a strong relationship between tumor purity and prognosis. In addition to the obvious differences in the immune microenvironment, the patients with high and low IRGS are also obviously different in terms of genomic alterations and enriched signaling pathways. This further highlights the potential significance of IRGS in prognostic risk stratification, and also provides an indicative value for revealing the molecular mechanisms in the context of prognostic differences. Additionally, as one of the key avenues of anti-tumor treatment, drugs to treat tumors have always been a hot topic of research. More and more drugs are being developed and used in clinical practice. Given the importance of these 8 IRGS-S genes in LUAD, we explored the relevance of these genes with FDA approved drugs. All eight genes were related to drugs, suggesting that these genes might be potential targets for drug therapy in LUAD. Among these, CD69 had potential associations with 41 drugs (e. g., nelarabine, bendamustine, asparaginase, ifosfamide, imatinib et al.). A previous study [79] has reported that the expression of CD69 might predict the response to bendamostine, its regulation by ibrutinib or idlisib could enhance the cytotoxic effects of chronic lymphocytic leukemia. This was in line with the findings from our study. Given the great advantages of IRGS in prognostic risk stratification, we attempted to explore its potential in predicting sensitivity to drug therapy. Our data indicate that patients with high IRGS present with increased TMB, and decreased TIDE, suggesting that patients with high IRGS are more likely to be the population to benefit from immunotherapy. In addition, we also found that the IC50 of several common chemotherapeutic drugs (Bortezomib,Cisplatin,Cyclopamine,Docetaxel,Epothilone.B, Paclitaxel, Parthenolide, Pazopanib) was lower in the high IRGS group, which also suggests that patients with high IRGS may benefit more from the application of chemotherapeutic drugs. It is worth noting that, in addition to cisplatin, docetaxel, Paclitaxel, other chemotherapeutic drugs such as Bortezomib and Cyclopamine have not been widely used in the treatment of lung cancer. Among these, Bortezomib is a reversible inhibitor of 26S proteasome-like activity in mammalian cells. Data from previous studies [80, 81] suggested that Bortezomib may have the greatest clinical benefit when used in combination with other therapies. Single-agent bortezomib causes growth inhibition and apoptosis in many NSCLC cell lines in vitro, and has antitumor activity in vivo [81]. Cyclopamine, the first compound found to inhibit Hedgehog signaling, binds to the Smo protein, thereby inhibiting its activity. Cyclopamine showed antitumor activity in multiple tumors [82]. It has been shown that Cyclopamine causes a significant reduction in oxygen consumption in many NSCLC cell lines, inhibiting NSCLC cell proliferation and inducing apoptosis. Cyclopamine also increases ROS production, mitochondrial membrane hyperpolarization, and mitochondrial breakage, thereby disrupting mitochondrial function in NSCLC cells [83]. These FDA-approved drugs have also shown antitumor activity. However, their application in lung cancer is still slow, and a large number of clinical studies are needed to further validate them in the future.

In conclusion, this study comprehensively analyzed the inflammatory response genes and identified three distinct tumor molecular subtypes with obviously different immune characteristics in LUAD. Besides, based on inflammatory response genes, we established a scoring tool called IRGS, which was also strongly correlated with immune infiltration and genomic landscape in LUAD and displayed the potential in predicting drug therapeutic responses. Moreover, we described the multi-dimensional characterization of 8 IRGS-S genes in LUAD, and emphasized the nonnegligible roles the inflammatory response genes played in shaping individual TME and in directing therapeutic intervention plans for LUAD.

## Supplementary Information

**Additional file 1. Figure S1**. Acquisition of candidate genes and their expression patterns across three tumor molecular subtypes. (**A**) KEGG analysis revealed the enriched pathways for up-regulated genes in tumor tissues. (**B**) KEGG analysis revealed the enriched pathways for down-regulated genes in tumor tissues. (**C**) Forest map shows the correction between the candidate genes and prognosis. (**D**) Heatmap shows that expression patterns of 24 prognosis-related IRGs among distinct tumor molecular subtypes. The Inf_Clusters (3 molecular subtypes), age, sex, EGFR mutation, N, T, TNM stage were used as patient annotations. **Figure S2**. The biological characteristics and immune infiltration across the three tumor molecular subtypes. (**A**, **B**) Unsupervised clustering for 24 prognosisrelated IRGs in TCGA cohort with cluster number 3. (**C**) GSVA enrichment analysis showing the activation states of biological pathways in distinct tumor molecular subtypes (Inf-Cluster C vs B). The heatmap was used to visualize these biological processes, and MediumVioletRed represented activated pathways and SteelBlue represented inhibited pathways. The TCGA-LUAD cohort was used as a sample annotation. (**D**) Immune infiltration characteristics of the three tumor molecular subtypes based on ssGSEA algorithm. The asterisks represented the statistical p value (*P < 0.05; **P < 0.01; ***P < 0.001). **Figure S3**. Tumor molecular subtype-related DEGs and enriched biological pathways. (**A**) Overlapping differentially expressed genes (DEGs) among the three tumor molecular subtypes. (**B**) KEGG enrichment analysis revealed that these overlapping genes were primarily involved in activities such as focal adhesion, phagosomes, NOD-like receptor signaling, and regulation of the actin cytoskeleton. (**C**) Gene Ontology analysis uncovered the biological activities these overlapping genes involved in biological processes. **Figure S4**. Subgroup analyses and the Kaplan-Meier survival curves were performed to verify the predictive performance of the IRGS in the different LUAD subgroups. **Figure S5**. The expression of these IRGS-S genes in tumor versus normal tissues from the protein level. These analyses were derived from the UALCAN (ualcan.path.uab. edu/analysis-prot.html) and HPA (https://www.proteinatlas.org/). **Figure S6**. Association between CNV and gene expression.

**Additional file 2. Table S1**. The list of immune signatures gene sets.

**Additional file 3. Table S2**. GSVA enrichment analysis showing the activation states of biological pathways in distinct tumor molecular subtypes.

**Additional file 4. Table S3**. The overlapping differentially expressed genes (DEGs) among the three tumor molecular subtypes.

**Additional** **file 5. Table S4**. Multivariate Cox analysis (stepwise regression models) for the construction of an IRGS.

**Additional file 6. Table S5**. The correlation of FDA approved drugs Z scores with the IRG expression values.

**Additional** **file 7. Table S6**. Analysis of differential gene expression between high and low IRGS groups.

## Data Availability

The datasets generated and analysed during the current study are publicly available in the TCGA repository (https://portal.gdc.cancer.gov), GEO repository (https://www.ncbi.nlm.nih.gov/geo/), GTEx repository (https://commonfund.nih.gov/GTEx/), UCSC Xena repository (https://xenabrowser.net), and CellMiner repository (https://discover.nci.nih.gov/cellminer/home.do).

## Abbreviations

LUAD: Lung adenocarcinoma
NSCLC: Non-small cell lung cancer
TME: Tumor microenvironment
IRGs: Inflammatory response genes
IRGS: Inflammatory response gene score
TCGA: The cancer genome atlas
GEO: Gene expression omnibus
GSEA: Gene-set enrichment analysis
GTEx: Genome tissue expression database
CNV: Copy number variation
DEGs: Differentially expressed genes
ROC: Receiver operating characteristic curve
AUC: Area under the curve
ssGSEA: Single sample GSEA
ESTIMATE: The estimation of stromal and immune cells in malignant tumors using expression data
GSVA: Gene set variation analysis
IC50: Semi-inhibitory concentration
TILs: Tumor infiltrating lymphocytes
CYT: Cytolytic activity
TIDE: Tumour immune dysfunction and exclusion
