# Supplementary material for: Comprehensive analysis reveals the potential value of inflammatory response genes in the prognosis, immunity, and drug sensitivity of lung adenocarcinoma
Source: BMC Med Genomics. 2022 Sep 18;15:198. doi: 10.1186/s12920-022-01340-7 (PMC9484176; doi:10.1186/s12920-022-01340-7)
Supplement: Supplementary file 1 — Additional file 1. Figure S1. Acquisition of candidate genes and their expression patterns across three tumor molecular subtypes. (A) KEGG analysis revealed the enriched pathways for up-regulated genes in tumor tissues. (B) KEGG analysis revealed the enriched pathways for down-regulated genes in tumor tissues. (C) Forest map shows the correction between the candidate genes and prognosis. (D) Heatmap shows that expression patterns of 24 prognosis-related IRGs among distinct tumor molecular subtypes. The Inf_Clusters (3 molecular subtypes), age, sex, EGFR mutation, N, T, TNM stage were used as patient annotations. Figure S2. The biological characteristics and immune infiltration across the three tumor molecular subtypes. (A, B) Unsupervised clustering for 24 prognosisrelated IRGs in TCGA cohort with cluster number 3. (C) GSVA enrichment analysis showing the activation states of biological pathways in distinct tumor molecular subtypes (Inf-Cluster C vs B). The heatmap was used to visualize these biological processes, and MediumVioletRed represented activated pathways and SteelBlue represented inhibited pathways. The TCGA-LUAD cohort was used as a sample annotation. (D) Immune infiltration characteristics of the three tumor molecular subtypes based on ssGSEA algorithm. The asterisks represented the statistical p value (*P < 0.05; **P < 0.01; ***P < 0.001). Figure S3. Tumor molecular subtype-related DEGs and enriched biological pathways. (A) Overlapping differentially expressed genes (DEGs) among the three tumor molecular subtypes. (B) KEGG enrichment analysis revealed that these overlapping genes were primarily involved in activities such as focal adhesion, phagosomes, NOD-like receptor signaling, and regulation of the actin cytoskeleton. (C) Gene Ontology analysis uncovered the biological activities these overlapping genes involved in biological processes. Figure S4. Subgroup analyses and the Kaplan-Meier survival curves were performed to verify the predictive performa [file 12920_2022_1340_MOESM1_ESM.pdf]

Comprehensive analysis reveals the potential value of inflammatory response genes in the prognosis, immunity, and drug sensitivity of lung adenocarcinoma

Congkuan Song#, Shize Pan#, Donghang Li, Bo Hao, Zilong Lu, Kai Lai, Ning Li and Qing Geng*

Department of Thoracic Surgery, Renmin Hospital of Wuhan University, Wuhan, China.

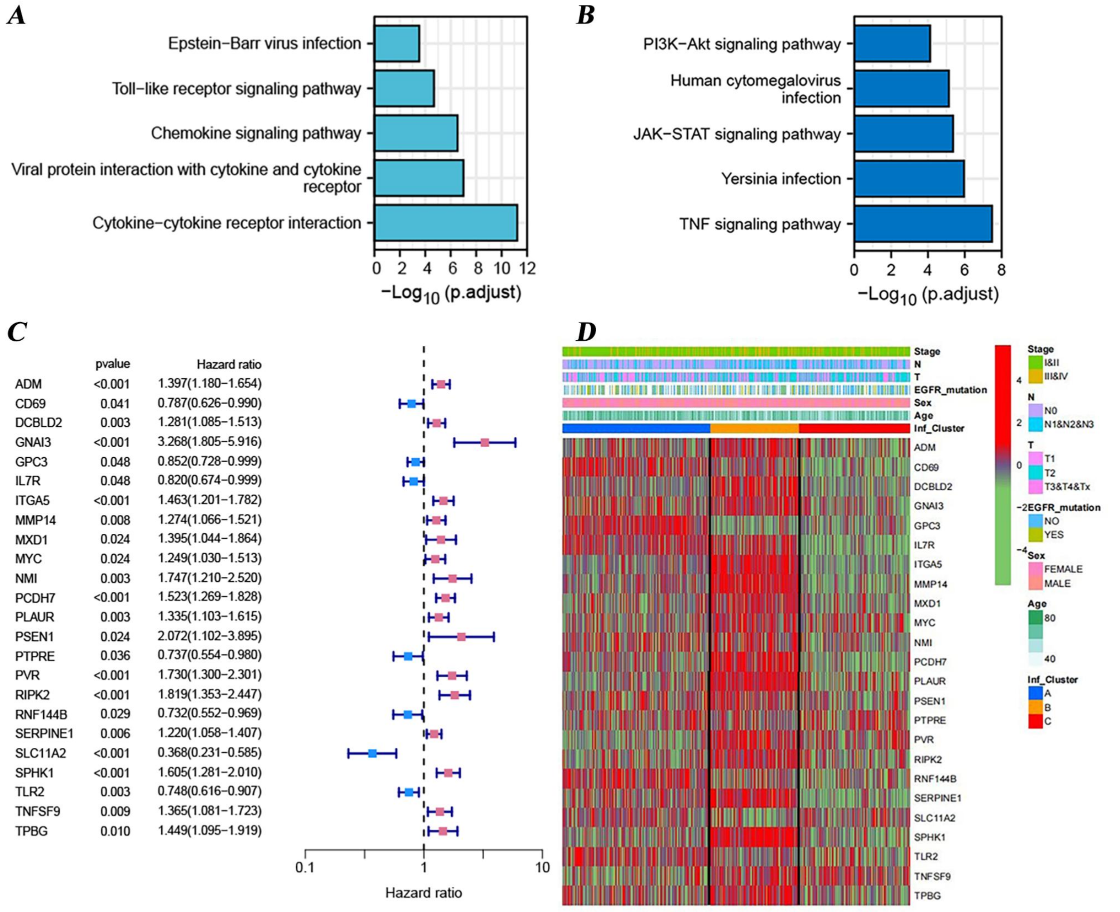

Figure S1. Acquisition of candidate genes and their expression patterns across three tumor molecular subtypes. (A) KEGG analysis revealed the enriched pathways for up-regulated genes in tumor tissues. **(B)** KEGG analysis revealed the enriched pathways for down-regulated genes in tumor tissues. **(C)** Forest map shows the correction between the candidate genes and prognosis. **(D)** Heatmap shows that expression patterns of 24 prognosis-related IRGs among distinct tumor molecular subtypes. The Inf_Clusters (3 molecular subtypes), age, sex, EGFR mutation, N, T, TNM stage were used as patient annotations.

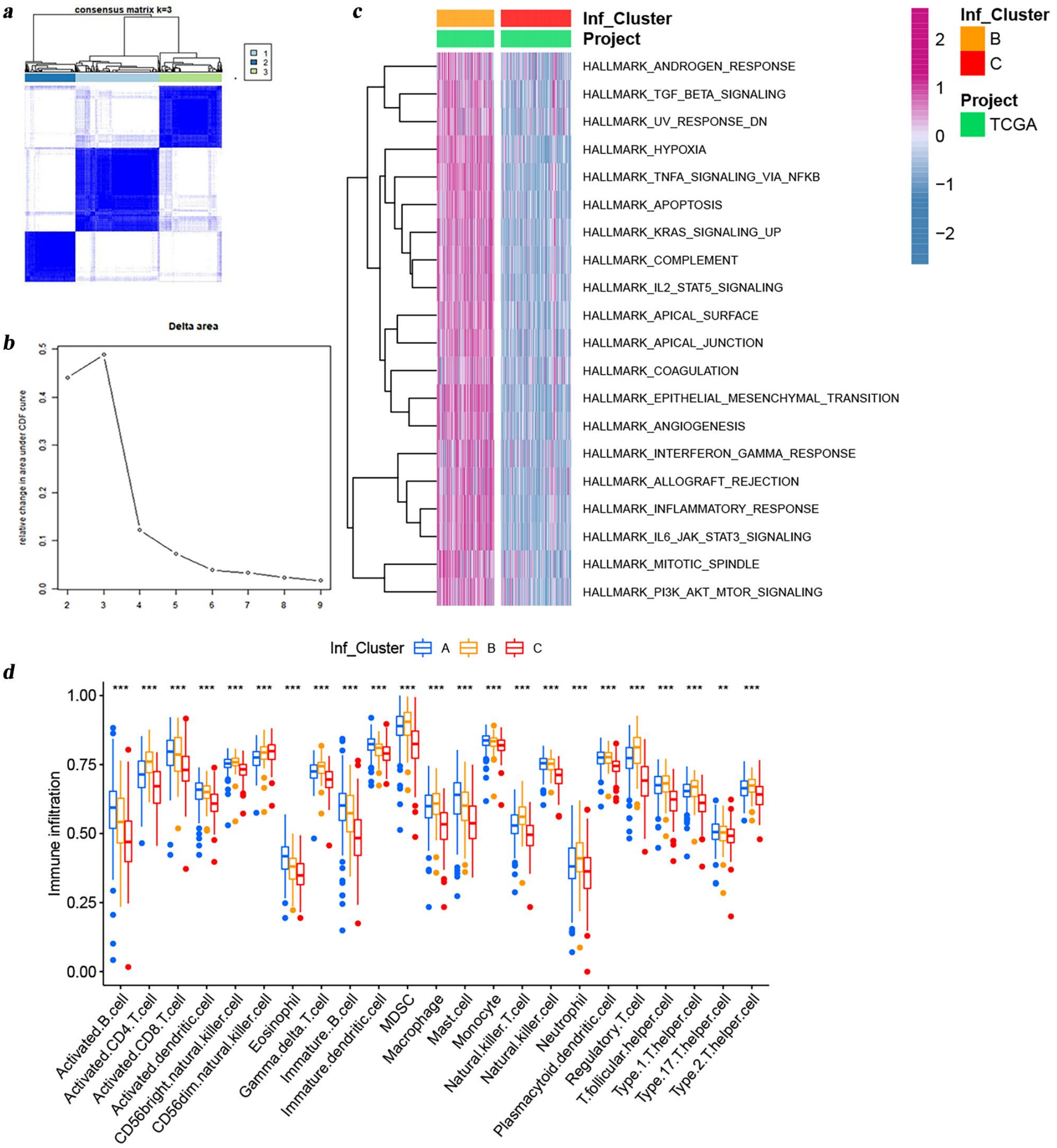

Figure S2. The biological characteristics and immune infiltration across the three tumor molecular subtypes. (A-B) Unsupervised clustering for 24 prognosis-related IRGs in TCGA cohort with cluster number 3. **(C)** GSVA enrichment analysis showing the activation states of biological pathways in distinct tumor molecular subtypes (Inf-Cluster C vs B). The heatmap was used to visualize these biological processes, and MediumVioletRed represented activated pathways and SteelBlue represented inhibited pathways. The TCGA-LUAD cohort was used as a sample annotation. **(D)** Immune infiltration characteristics of the three tumor molecular subtypes based on ssGSEA algorithm. The asterisks represented the statistical p value (*P < 0.05; **P < 0.01; ***P < 0.001).

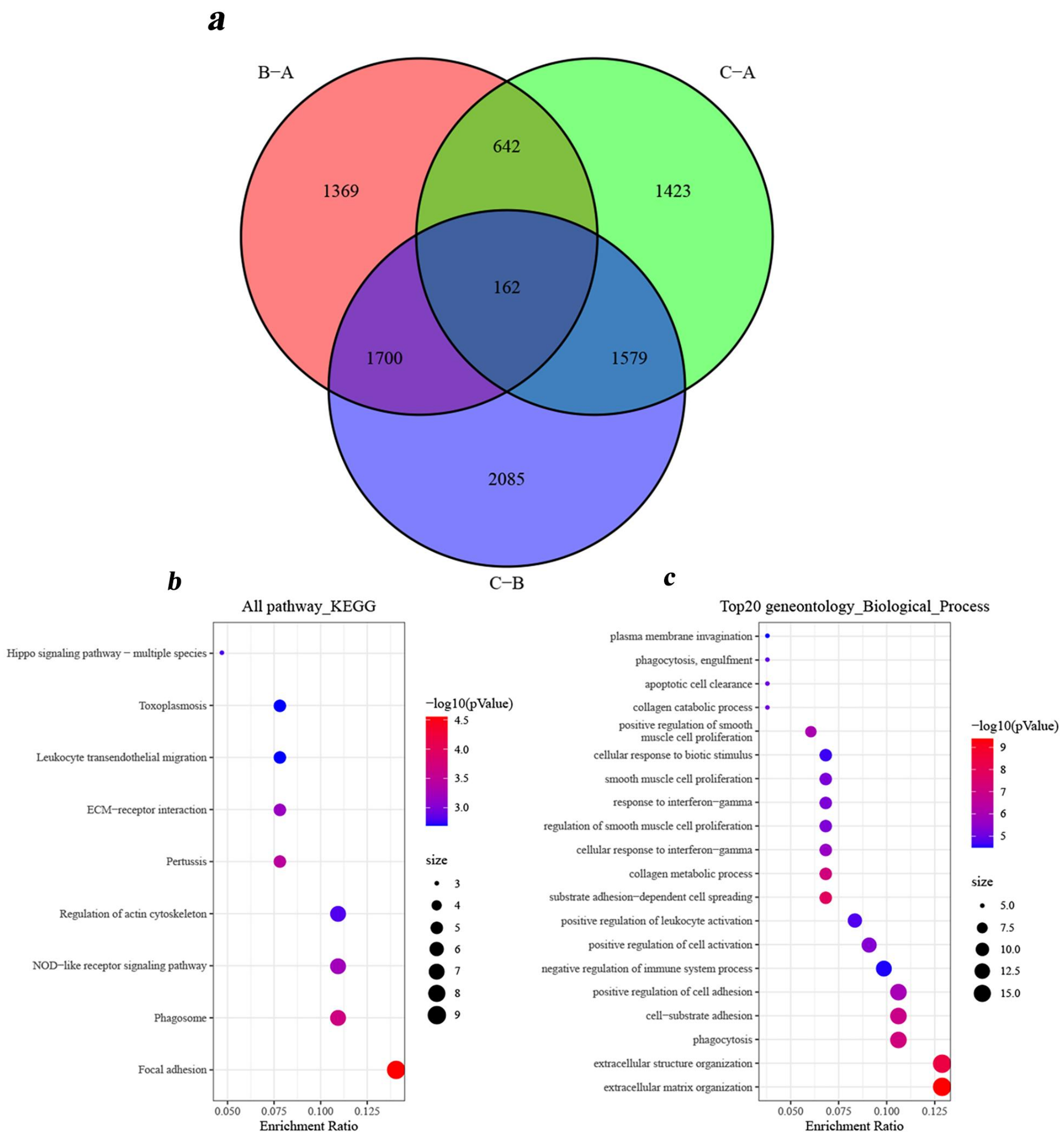

Figure S3. Tumor molecular subtype-related DEGs and enriched biological pathways. (A) Overlapping differentially expressed genes (DEGs) among the three tumor molecular subtypes. **(B)** KEGG enrichment analysis revealed that these overlapping genes were primarily involved in activities such as focal adhesion, phagosomes, NOD-like receptor signaling, and regulation of the actin cytoskeleton. **(C)** Gene Ontology analysis uncovered the biological activities these overlapping genes involved in biological processes.

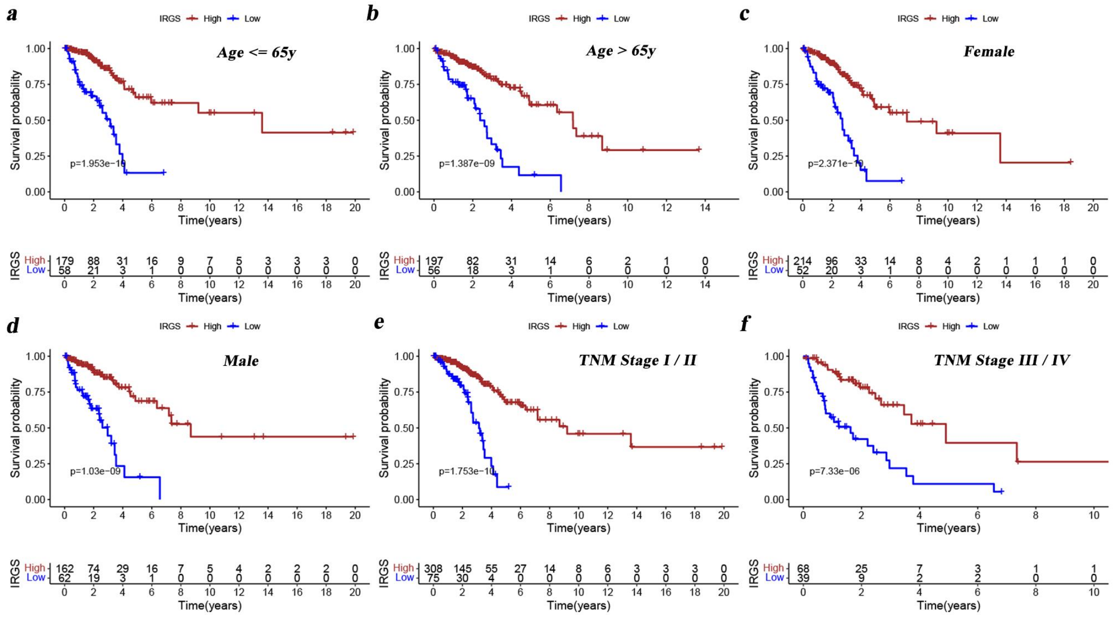

Figure S4. Subgroup analyses and the Kaplan-Meier survival curves were performed to verify the predictive performance of the IRGS in the different LUAD subgroups.

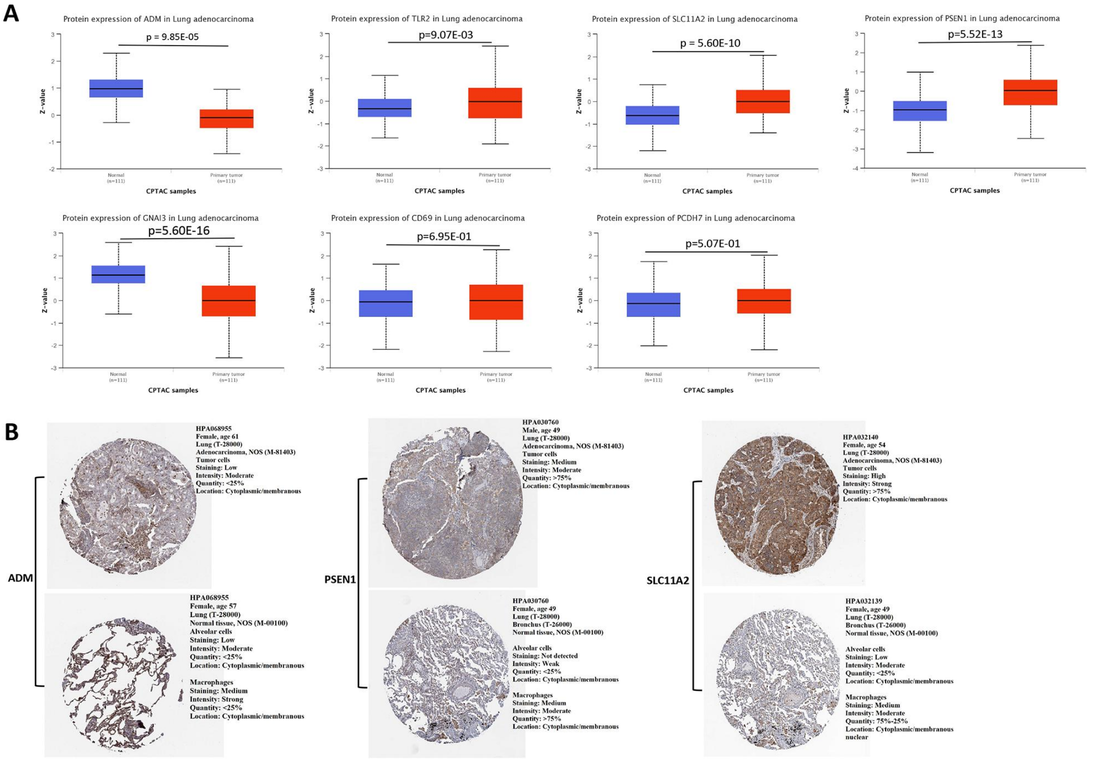

Figure S5. The expression of these IRGS-S genes in tumor versus normal tissues from the protein level. These analyses were derived from the UALCAN (ualcan.path.uab. edu/analysis-prot.html) and HPA (https://www.proteinatlas.org/).

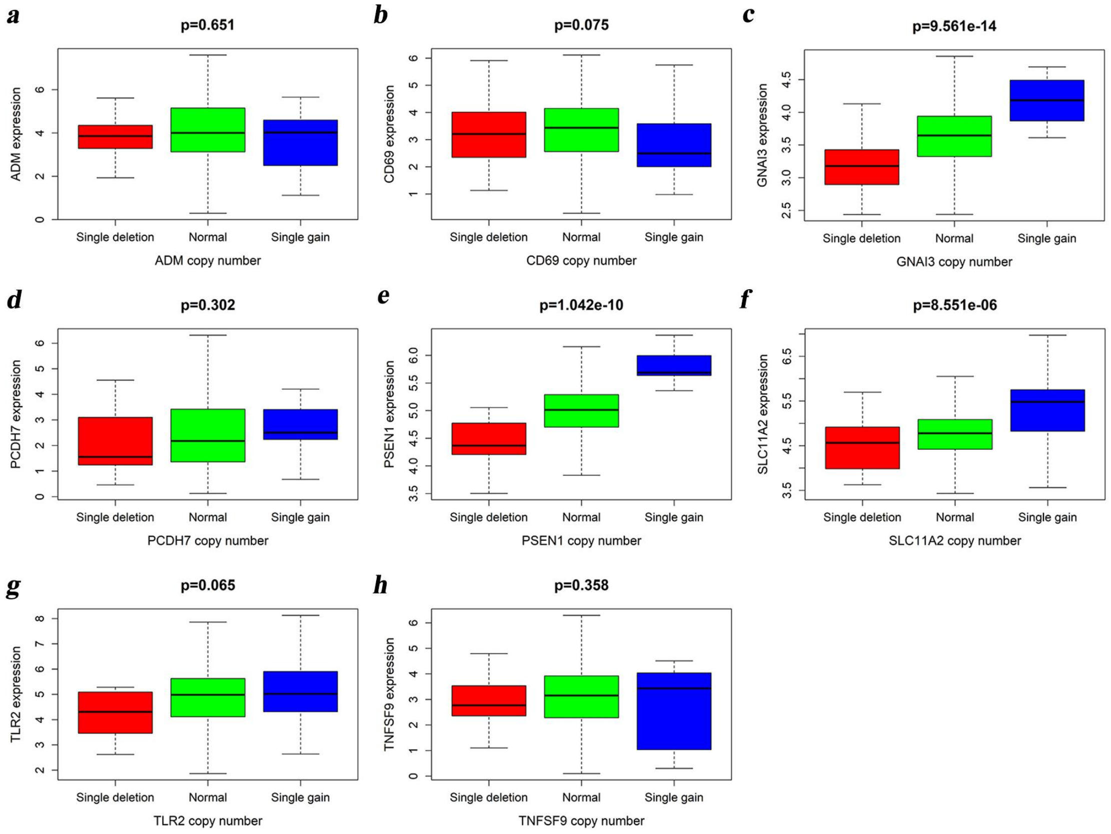

Figure S6. Association between CNV and gene expression.
